# Evaluation of Anterior Segment Parameters in Pseudoexfoliative Glaucoma, Primary Angle-Closure Glaucoma, and Healthy Eyes

**DOI:** 10.4274/tjo.03271

**Published:** 2018-10-31

**Authors:** Nilgün Özkan Aksoy, Burçin Çakır, Emine Doğan, Gürsoy Alagöz

**Affiliations:** 1 Sakarya University Training and Research Hospital, Department of Ophthalmology, Sakarya, Turkey

**Keywords:** Pseudoexfoliative glaucoma, primary angle-closure glaucoma, dual Scheimpflug topography system

## Abstract

**Objectives:**

To evaluate anterior segment parameters measured by dual Scheimpflug corneal topography in pseudoexfoliative glaucoma (PEXG), primary angle-closure glaucoma (PACG), and healthy eyes.

**Materials and Methods:**

One hundred forty-three eyes of 86 patients were included in this study. Forty-seven eyes of 38 patients with PEXG, 30 eyes of 15 patients with PACG, and 66 eyes of 33 healthy subjects were evaluated. Patients who underwent previous ophthalmic surgery and contact lens wearers were excluded. After full ophthalmological examination, mean central corneal thickness (CCT), white-to-white horizontal corneal diameter (WTW), pupillary diameter (PD), anterior chamber volume (ACV), anterior chamber depth (ACD), and mean anterior chamber angle were measured by dual Scheimpflug corneal topography and compared between the three groups. Statistical analyses were done using Statistical Package for Social Sciences for Windows 18.0 program.

**Results:**

No statistical difference was found in mean age or gender among the study groups (p>0.05). There were also no statistical differences in CCT, WTW, or PD among the groups (p=0.568, p=0.064, p=0.321, respectively). ACV, ACD, and mean anterior chamber angle values were significantly lower in the PACG group compared to the other groups (p=0.000 for all). There was no statistically significant difference in these measurements between the PEXG and normal eyes.

**Conclusion:**

ACV and depth and mean anterior chamber angle were statistically different (lower) in PACG when compared with PEXG and healthy eyes. Dual Scheimpflug corneal topography can be used as an objective method for the measurement of anterior segment parameters in glaucoma.

## Introduction

Pseudoexfoliative glaucoma (PEXG) is a type of secondary glaucoma which is characterized by the production and accumulation of abnormal extracellular fibrillar material in the lens capsule, iris, non-pigmented ciliary epithelium, trabecular meshwork, and corneal endothelial cells. This accumulation causes intraocular complications including cataract, open-angle glaucoma, angle-closure glaucoma, lens decentration, and iridopathy.^1,2^

Primary angle-closure glaucoma (PACG) is a major blinding form of glaucoma in Asia.^3^ The two main mechanisms of the disease are pupillary block and plateau iris syndrome. Besides these, anatomical differences in the iris, lens, and ciliary body have also been shown to play important roles in the pathogenesis. Shallow anterior chamber, thicker lens, anterior lens position, smaller corneal diameter, and anterior displacement of the lens-iris diaphragm are biometric characteristics of PACG.^3,4^

Intraocular pressure is an independent risk factor for glaucomatous progression and its measurement is affected by central corneal thickness (CCT). Therefore, we may say that CCT is associated with glaucoma because of its effect on tonometry. Ultrasonic pachymetry is widely used to measure CCT, but this method has some disadvantages. The accuracy and repeatability of measurements are dependent on accurate placement of the probe on the cornea. In addition, corneal indentation may result in an underestimated CCT value.^5,6^ As a result, non-contact techniques are needed for the assessment of CCT. Prior studies have shown that highly reproducible CCT measurements can be obtained using dual Scheimpflug imaging systems.^7,8,9^ Detailed anterior chamber angle (ACA) evaluation is essential for the diagnosis of PACG and PEXG. Gonioscopy is the gold standard technique for this evaluation. However, this technique requires a contact lens, topical anesthesia, and an experienced examiner to provide a confident diagnosis. Anterior segment imaging devices may be beneficial as a useful, non-contact method for angle closure screening. The parameters obtained with dual Scheimpflug imaging have been shown to correlate well with gonioscopy.^10^ Anterior chamber depth (ACD) and anterior chamber volume (ACV) measurements are also important in both PACG and PEXG.^11,12^

The dual Scheimpflug imaging system is the basis for a number of devices that can image the anterior segment. It allows for photographic documentation of the anterior segment with a depth of focus ranging from the anterior cornea to the posterior lens surface. It is capable of estimating ACD, ACV, and ACA.^13^

In this study, we aimed to evaluate anterior segment parameters measured using the Galilei G4 Dual Scheimpflug Analyzer imaging device (Ziemer Ophthalmic Systems AG, Switzerland) in patients with PEXG and PACG and to compare these groups with healthy subjects.

## Materials and Methods

This cross-sectional study was conducted at the Sakarya University Department of Ophthalmology. Prior approval was obtained from the Institutional Review Board (71522473/050.01.04/194) and written informed consent was obtained from each subject. The study was performed in adherence to the Declaration of Helsinki. Forty-seven eyes of 38 patients with PEXG (group 1), 30 eyes of 15 patients with PACG (group 2), and 66 eyes of 33 healthy subjects (group 3) were examined in this study. 

Inclusion criteria for group 1 were high intraocular pressure (over 21 mmHg), visible pseudoexfoliation material on the anterior segment structures, glaucomatous optic nerve head changes (notching of optic disc rim, higher vertical cup-to-disc ratio, retinal nerve fiber layer hemorrhages), and glaucomatous visual field defects (scotomas indicating loss of the nerve fiber layer) detected by computerized visual field examination. Group 2 included patients with high intraocular pressure (over 21 mmHg), narrow ACA detected by gonioscopy, glaucomatous optic nerve head changes (notching of optic disc rim, higher vertical cup-to-disc ratio, retinal nerve fiber layer hemorrhages), glaucomatous visual field defects (scotomas indicating loss of the nerve fiber layer), and no history of laser peripheral iridotomy. Inclusion criteria for group 3 were normal intraocular pressure (under 21 mmHg) and no abnormal findings in anterior segment, fundus, or visual field examinations. Patients with corneal pathology (dry eye, keratoconus, history of contact lens use), uveitis, previous ocular surgery, history of contact lens use, previous ocular trauma, posterior segment pathology (retinal and optic nerve diseases which might affect visual field tests and retinal nerve fiber layer), and refractive errors greater than ±3 diopters were excluded from all groups.

All patients underwent full ophthalmic examination including best corrected visual acuity measured by Snellen chart, intraocular pressure measurement with Goldmann applanation tonometry, and detailed dilated fundus examination. In addition, Humphrey 30-2 SITA FAST visual field test and spectral-domain optical coherence tomography (SD-OCT) were performed.

The anterior segment was evaluated using a Galilei G4 Dual Scheimpflug Analyzer (Ziemer Ophthalmic Systems AG, Switzerland). Measurements were performed under scotopic conditions with undilated pupils by the same ophthalmologist (N.Ö.A). Mean ACA, ACD, ACV, CCT, pupil diameter, and horizontal white-to-white (WTW) corneal diameter values were obtained.

### Statistical Analysis

Statistical analysis was performed with SPSS for Windows version 18 (SPSS Inc, Chicago, IL, USA). All data were reported as means and standard deviation. Normality of continuous variables within the groups was determined by Shapiro-Wilk test. Chi-square test and ANOVA tests were used. A p value <0.05 was considered statistically significant.

## Results

The demographic features of the three groups are summarized in Table 1. There was no statistically significant difference between the groups with respect to age or gender (p>0.05).

Mean ACA, ACD, ACV, pupil diameter, WTW corneal diameter, and CCT values of the three groups are shown in Table 2. Mean ACV and ACD were significantly lower and mean ACA was significantly narrower in the PACG group (group 2). There were no significant differences with respect to pupil diameter, WTW corneal diameter, or CCT among three groups. Mean CCT was markedly thinner in the PACG group (group 2) compared to the control group (group 3), but this difference was not statistically significant.

The patients in groups 1 and 2 used antiglaucomatous agents including prostaglandin analogues.

## Discussion

Anterior chamber parameters such as ACD, ACV, and ACA have an important role in the diagnosis and evaluation of every type of glaucoma. Evaluation of the ACA is essential in glaucoma patients that can be subjectively evaluated with the Shaffer and Van Herick methods or gonioscopy. Different quantitative methods such as ultrasonic biomicroscopy, OCT, and Orbscan provide repeatable, accurate ACA measurements. Several studies have measured ACA and other anterior segment parameters in healthy and glaucomatous eyes using different methods.^12,14,15,16,17,18^

Pakravan et al.^14^ evaluated anterior segment parameters in the unaffected fellow eyes of subjects with a previous episode of PACG using Pentacam and identified eyes at high risk of PACG among primary angle closure suspects. They claimed that ACV, ACA, and ACD are probably powerful indicators for determining the risk of acute angle closure (AAC) with cutoff values of ACV ≤100 µL, ACA ≤26°, and ACD ≤2.1 mm. Our findings in PACG subjects are consistent with their study. 

Various parameters obtained with dual Scheimpflug imaging devices correlate well with gonioscopy.^13^ However, ACA measurement by dual Scheimpflug devices may not be accurate because the entire angle is not fully visible due to total internal reflection. The correlation between ACA measurements and gonioscopic grade is better with anterior segment OCT (AS-OCT) and ultrasound biometry when compared to dual Scheimpflug.^19^ Kurita et al.^11^ compared Pentacam and ultrasound biomicroscopy and reported that Pentacam effectively measured ACD and ACV in eyes with PACG and PACG suspects, but not ACA. They reported that Pentacam ACA measurements were not reliable when evaluating eyes with a Shaffer grade of 2 or less. Grewal et al.^10^ compared Pentacam and AS-OCT and reported that ACV had the highest discriminating ability (AUC=0.935) in detecting narrow angles. The Pentacam cannot directly visualize the angle; the breadth of three-dimensional data incorporated in its analyses is its disadvantage. In contrast, non-contact AS-OCT assessment limited to cross-sections of only the nasal and temporal angles may exclude representative information regarding the angle. To image the superior and inferior angles, contact would be required to move the eyelids obscuring visualization.^19^In a recent report, it was noted that non-contact imaging using OCT, dual Scheimpflug photography, or scanning peripheral ACD analyzer is superior to contact imaging using ultrasound biomicroscopy for large-scale primary angle closure screening.^20^

The high incidence of narrow angle configuration observed in patients with pseudoexfoliation may be associated with increased iris thickness, posterior synechiae, and zonular weakness. Doganay et al.^12^ reported that the mean ACD measurement in patients with PEXG patients was found to be shallower than in healthy individuals. However, they found no statistical difference in ACD between PEXG and pseudoexfoliation syndrome. They also reported that there were no significant differences in ACV, ACA, or CCT parameters among patients with pseudoexfoliation syndrome, those with PEXG, and healthy controls.^12^ Guneş et al.^15^ evaluated anterior segment parameters in patients with pseudoexfoliation syndrome using dual Scheimpflug imaging and reported that there were no significant differences in ACA, ACD, or ACV values. Similarly, there were no statistically significant differences in ACA, ACD, or ACV between the patients with PEXG and the control group in our study.

Central corneal thickness is an important parameter in eyes with glaucoma. Studies evaluating differences in CCT among glaucoma types were performed previously. Some of these studies did not find any significant difference in CCT between PEXG and primary open-angle glaucoma (POAG).^16,17,18^ Kitsos et al.^21^, Bechmann et al.^22^, Gorezis et al.^23^, and Kniestedt et al.^24^ found CCT to be significantly lower in PEXG compared to POAG. Pang et al.^25^ and Tolesa and Gessesse^26^ found no significant difference in CCT between PACG and POAG eyes, but Moghimi et al.^27^ found thicker CCT in PACG than in normal healthy eyes. This variation in results could be due to differences in measurement methods, sample sizes, and ethnicities. In our study, there was no significant difference in CCT among groups. 

Prostaglandin analogues have biological effects on extracellular matrix and collagen metabolism.^28^ Altan et al.^29^ revealed that CCT was reduced with the use of 0.005% latanoprost, while ACD was not affected. In our study, patients were not classified according to antiglaucomatous medications used. This is a limitation of our study. 

Dual Scheimpflug systems are able to provide highly repeatable CCT measurements.^5,7,8^ In some studies, no difference has been observed in mean CCT obtained by ultrasound pachymetry or Pentacam.^6,30^ In contrast, several other studies have revealed significant differences in mean CCT values measured by Pentacam and ultrasound pachymetry.^31,32^ Although these differences may be small, comparing CCT values across different measurement platforms is not advised. Prior studies have shown that highly reproducible CCT measurements can be obtained by the Pentacam, Sirius, Galilei, and Corvis ST. Of these devices, the Galilei has the highest reported intraoperator repeatability. This may be due to its dual-rotating camera design, which can average the CCT estimated from two different dual Scheimpflug cameras.^5^ In our study, we used Galilei for measuring CCT.

## Conclusion

In conclusion, mean ACV, ACD, and ACA values measured with dual Scheimpflug imaging system were found to differ significantly in the PACG group. There were no statistically significant differences in anterior segment parameters between the PEXG group and healthy eyes. Therefore, dual Scheimpflug corneal topography can be used as an objective measurement method for anterior segment parameters in glaucoma.

## Figures and Tables

**Table 1:**
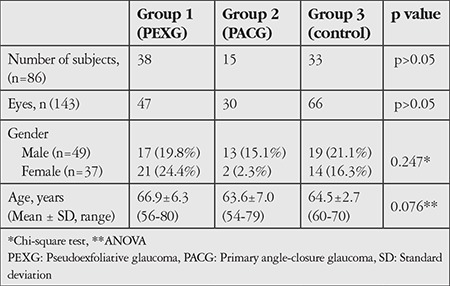
Comparison of demographic data of patients in the pseudoexfoliative glaucoma, primary angle-closure glaucoma, and healthy control groups

**Table 2:**
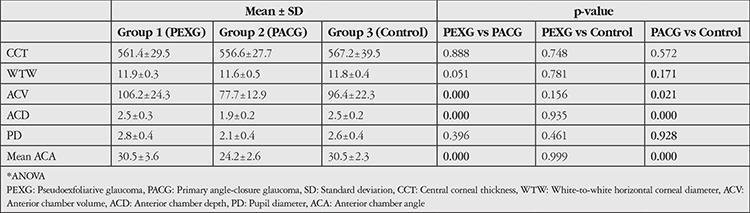
Comparison of anterior segment parameters in eyes with pseudoexfoliative glaucoma, eyes with primary angle-closure glaucoma, and healthy controls
